# Association between an oxidative balance score and mortality: a prospective analysis in the SUN cohort

**DOI:** 10.1007/s00394-023-03099-8

**Published:** 2023-02-12

**Authors:** Irene Talavera-Rodriguez, Cesar I. Fernandez-Lazaro, Ángela Hernández-Ruiz, Maria S. Hershey, Cristina Galarregui, Mercedes Sotos-Prieto, Carmen de la Fuente-Arrillaga, Miguel Ángel Martínez-González, Miguel Ruiz-Canela

**Affiliations:** 1 Department of Preventive Medicine and Public Health, School of Medicine, University of Navarra, C/ Irunlarrea, 31008 Pamplona, Spain; 2 IdisNA, Navarra Institute for Health Research, 31008 Pamplona, Spain; 3 Iberoamerican Nutrition Foundation (FINUT), Armilla, 18016 Granada, Spain; 4 Department of Nutrition, Food Sciences and Physiology and Centre for Nutrition Research, Faculty of Pharmacy and Nutrition, University of Navarra, 31008 Pamplona, Spain; 5 Department of Preventive Medicine and Public Health, School of Medicine, Universidad Autónoma de Madrid, 28029 Madrid, Spain; 6 CIBER of Epidemiology and Public Health (CIBERESP), Carlos III Health Institute, 28029 Madrid, Spain; 7 Department of Environmental Health, Harvard T.H. Chan School of Public Health, Boston, MA 02115 USA; 8 IMDEA-Food Institute. CEI UAM+CSIC, Ctra. de Canto Blanco 8, E. 28049, Madrid, Spain; 9 CIBER of Physiopathology of Obesity and Nutrition (CIBEROBN), Carlos III Health Institute, 28029 Madrid, Spain; 10 Department of Nutrition, Harvard T.H. Chan School of Public Health, Harvard University, Boston, MA 02115 USA

**Keywords:** Oxidative balance score, Oxidative stress, Mortality, Diet, Lifestyle, Dietary antioxidants

## Abstract

**Purpose:**

We aimed to prospectively investigate the association of an overall oxidative balance score (OBS) with all-cause death and cause-specific mortality among participants in the Seguimiento Universidad de Navarra (SUN) Study, a Mediterranean cohort of Spanish graduates.

**Methods:**

Using baseline information on 12 a priori selected dietary and non-dietary lifestyle pro- and antioxidants exposures—vitamins C and E, β-carotenes, selenium, zinc, heme iron, polyphenols, total antioxidant capacity, body mass index, alcohol, smoking, and physical activity—we constructed an equally weighted OBS categorized into quartiles, with higher scores representing greater antioxidant balance. Cox proportional hazards models were fitted to evaluate the association between the OBS and mortality.

**Results:**

A total of 18,561 participants (mean [SD] age, 38.5 [12.4] years; 40.8% males) were included in the analysis. During a median follow-up of 12.2 years (interquartile range 8.3–14.9), 421 deaths were identified, including 80 deaths from cardiovascular disease (CVD), 215 from cancer, and 126 from other causes. After adjustment for potential confounders, the hazard ratios and 95% confidence interval (CIs) between the highest quartile (predominance of antioxidants) *vs.* the lowest quartile (reference category) were 0.35 (95% CI 0.22–0.54,* P*-trend < 0.001) for all-cause mortality, 0.18 (95% CI 0.06–0.51,* P*-trend = 0.001) for CVD mortality, 0.35 (95% CI 0.19–0.65,* P*-trend = 0.002) for cancer mortality, and 0.45 (95% CI 0.20–1.02,* P*-trend = 0.054) for other-cause mortality.

**Conclusion:**

Our findings suggest a strong inverse association between the OBS and all-cause, CVD, and cancer mortality. Individuals exposed to both antioxidant dietary and lifestyle factors may potentially experience the lowest mortality risk.

**Study registry number:**

Dynamic Mediterranean Prospective Cohort: the SUN Project; NCT02669602. https://clinicaltrials.gov/ct2/show/NCT02669602. https://proyectosun.es

**Supplementary Information:**

The online version contains supplementary material available at 10.1007/s00394-023-03099-8.

## Introduction

Non-communicable diseases (NCDs) are currently the leading causes of death in the world. Approximately 41 million people (71% of all deaths globally) die each year due to NCDs [1]. Among NCDs, cardiovascular disease (CVD) and cancer are at the top of the list, accounting for 17.9 and 9.3 million deaths annually, respectively. Modifiable risk factors, such as a sedentary lifestyle, unhealthy diet, tobacco use, and harmful alcohol intake, have been associated with premature deaths [2, 3], highlighting opportunities to minimize premature mortality through lifestyle changes.

Oxidative stress—the imbalance between oxidative and anti-oxidative components that leads to oxidative damage [4]— has been postulated as the main mechanism for aging [5, 6]. The production of reactive oxygen and nitrogen species (RONS) may accelerate the development of aging-related health outcomes, including various chronic diseases [7, 8] and mortality [9, 10]. Exogenous modifiable factors, such as diet, lifestyle, and medications, are involved in the body’s oxidative balance [11]. Some dietary exposures, such as carotenoids (α-carotene, β-carotene, lutein/zeaxanthin, β-cryptoxanthin, and lycopene), glucosinolates, tocopherols, vitamins C and E, polyphenols, polyunsaturated fats (PUFAs), certain minerals (zinc, selenium, and calcium), and lifestyle exposures such as physical activity may have a powerful antioxidant capacity. Other factors including dietary fat, heavy metals, and smoking, have a pro-oxidant effect [12]. A combined measure of multiple pro- and antioxidant exposures can be a more accurate indicator of the human body oxidative balance rather than individual exposures. According to this premise, epidemiological studies have used a variety of oxidative balance scores (OBS) to account for both dietary and non-dietary lifestyle exposures [13], but only three studies have examined the association of OBS with mortality risk in prospective cohort studies [14–16], considering only dietary factors [14], or both dietary and non-dietary factors [15, 16]. However, these studies were limited to certain populations, such as male smokers [14], high CVD risk individuals [15], and older women [16], and it is well-known the interrelated link between smoking, CVD risk, aging, oxidative stress, and mortality. To our knowledge, no previous study has analyzed the association between a comprehensive OBS and mortality in a Mediterranean middle-aged population with a high educational level at low cardiovascular risk. It is well known the strong link between educational attainment and mortality risk [17, 18], and the health benefits of the Mediterranean diet on cardiovascular outcomes [19].

The main objective of this study was to prospectively investigate the association between an OBS—based on 12 a priori selected dietary and non-dietary lifestyle pro- and antioxidants exposures—with all-cause, cardiovascular, and cancer mortality risk among participants in the Seguimiento Universidad de Navarra (SUN) Study, a Mediterranean cohort of middle-aged Spanish adults.

## Methods

### Study population

The SUN Project (www.proyectosun.es) is a prospective, multipurpose, dynamic cohort of Spanish university graduates, consisting of a baseline questionnaire and biennial follow-up questionnaires. Briefly, the SUN cohort is composed of graduates from the University of Navarra as well as from other different Spanish universities, all aged 20 years and over [20–22]. The recruitment started in 1999 and the cohort aims to identify dietary and lifestyle determinants of non-communicable diseases. The SUN investigation has been approved by the Institutional Review Board of the University of Navarra on August 30, 2001, in line with the principles of the Declaration of Helsinki, and registered at clinicaltrials.gov (NCT02669602). Participants’ informed consent was given upon completion of the baseline questionnaire. Further explanation of the design and methods of the SUN study has been previously published [22].

**Fig. 1:**
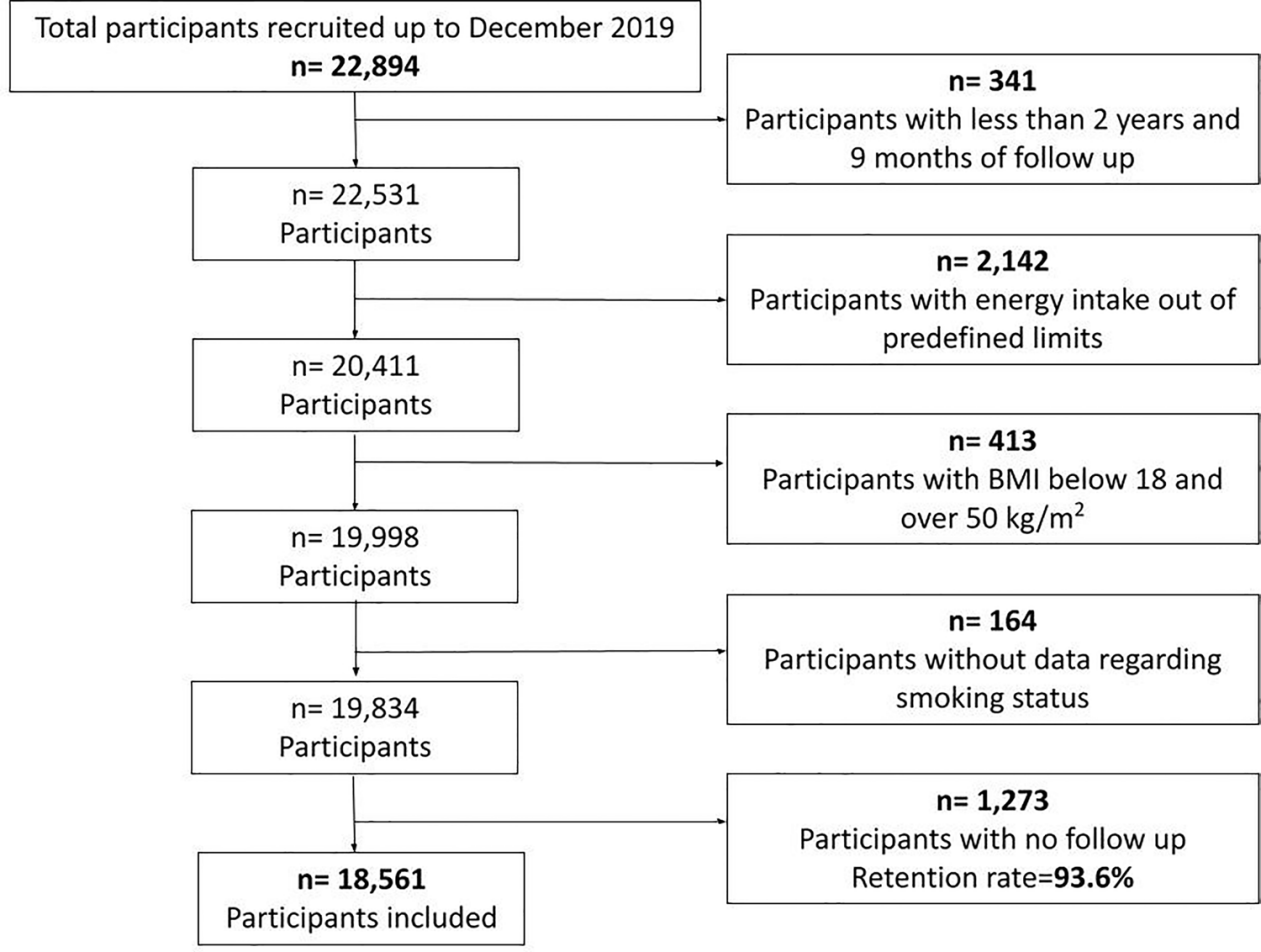
Flowchart of participants in the Seguimiento Universidad de Navarra Project, 1999 to 2019

From December 1999 until December 2019, a total of 22,894 participants were recruited. The exclusion criteria applied were: participants recruited < 2 years and 9 months since the end of the recruitment in the database (*n* = 341), participants with total daily energy intake out of predefined limits (men: 800–4,000 kcal/d, women: 500–3,500 kcal/d, [*n* = 2142]) according to published recommendations [23], participants with body mass index (BMI) below 18 or above 50 kg/m^2^ (*n* = 413), participants with missing data on smoking status (*n* = 164), and participants with no follow-up (*n* = 1273). A total of 18,561 participants (94% retention rate) were included in the final analysis (Fig. 1).

### The oxidative balance score (OBS)

The OBS was constructed using baseline dietary information from a semi-quantitative 136-item food-frequency questionnaire (FFQ) repeatedly validated in Spain [24–26]. Information about non-dietary lifestyle factors (i.e., smoking, alcohol intake, and physical activity) was collected from the baseline questionnaire. A previously validated questionnaire was used to evaluate physical activity [27] and self-reported weight and height measurements were validated in a cohort subgroup [28]. BMI was calculated by dividing weight by height squared (kg/m^2^). Regular use of multivitamins and supplements (brand, dosage, and frequency) was also collected.

This novel OBS was based on 12 a priori selected components including dietary and non-dietary lifestyle factors associated with oxidative balance, as either pro- or antioxidant exposures (Additional File 1: Table s1). The selection of these components was based on the latest existing evidence and information available from the FFQ and lifestyle questionnaire. Dietary intakes included total vitamin C, total vitamin E, beta-carotenes, selenium, total zinc, heme iron, polyphenol antioxidant content (PAC) score, and total antioxidant capacity (TAC), whereas lifestyle included BMI, alcohol intake, smoking status, and an 8-item physical activity score. Total intakes of vitamin C, vitamin E, and zinc included both diet and supplement intakes. The novelty of this OBS lies in the inclusion of supplementary intakes, and TAC and PAC components, which have not been previously included in other published OBS. PAC score was calculated based on a previous published score that assessed the polyphenol intake with a 7-item score by generating deciles of intakes for the included polyphenols class and subclass (flavonols, anthocyanins, flavanones, flavones, flavanols, isoflavonoids, and lignans intake) [29]. This scoring system was developed to overcome discrepancies in reported mean intakes of polyphenols across populations from different cohorts, consequence of methodological differences, like the use of different dietary assessment methods or food composition tables. TAC was calculated by computing the individual TAC values from the Ferric Reducing Antioxidant Power (FRAP) assays [30–33] for each item of the semi-quantitative FFQ, as previously reported [34]. The inclusion of TAC in our OBS provides a more holistic and complete approach of the global oxidative balance estimation, and it is particularly useful when some antioxidants are not provided in isolation, such as carotenoids [35]. Food composition tables were used to calculate nutrient and energy intake for each participant [36, 37]. Physical activity was assessed with a previous score that accounts for time and intensity of exercise as well as sedentary behaviors [38]. Each dietary component was categorized into quintiles, scoring 0 (first quintile) to 4 points (fifth quintile), except for heme iron which was inversely weighted (Additional File 1: Table s1). Each lifestyle component had a unique scoring criterion. BMI and alcohol intake were scored from 0 to 4 points using the following cut-off points: BMI ≥ 35, < 35 and ≥ 30, < 30 and ≥ 27, < 27 and ≥ 25, and < 25 kg/m^2^; alcohol intake for men, > 75, ≤ 75 and > 50, ≤ 50 and > 20, ≤ 20 and > 10, and ≤ 10 g/day, and for women, > 50, ≤ 50 and > 25, ≤ 25 and > 15, ≤ 15 and > 5, and ≤ 5 g/day. Smoking was scored 0, 2, or 4 points for current, former, and never smoking, respectively. Physical activity was also scored 0, 2, or 4 points for low, medium, and high, respectively [38]. The OBS was calculated as the sum of points for each component, for a total scoring range from 0 to 48 points, with higher scores indicating greater antioxidant balance. All the OBS components were equally weighted. Lastly, participants were categorized into quartiles of OBS.

### Outcome

The primary outcome of the study was all-cause mortality and secondary outcomes included CVD mortality, cancer mortality, and other-cause mortality. The SUN cohort keeps a close and permanent follow-up that provides continuously updated information about participants’ disease incidence and death. Deaths are reported by next of kin, work colleagues, and professional associations (such as alumni). In addition, we checked the Spanish National Death Index, and the National Statistics Institute (www.ine.es) at least once a year. All deaths were confirmed by death certificates and medical records. The date and cause of death were recorded and encoded using the Tenth Revision of the International Classification of Diseases (ICD-10).

#### Other covariates

Participants provided additional information at baseline on sociodemographic characteristics, medication use, personal and family history of medical conditions, and adherence to the Mediterranean diet (MedDiet) [39]. Participants with prevalent diabetes, dyslipidemia, CVD, hypertension, cancer, and depression were identified if they had a previous diagnosis and/or treatment for the respective condition. Prevalent CVD was defined as having at least one of the following events before entering the cohort: myocardial infarction, stroke, angina pectoris, coronary bypass, tachycardia, atrial fibrillation, aneurysm, cardiac insufficiency, pulmonary embolism, deep vein thrombosis, or intermittent claudication.

### Statistical analysis

Baseline characteristics for all participants and OBS components were described according to OBS quartiles, expressed as means with standard deviations for numerical variables or percentages for categorical variables. Pearson product–moment correlation coefficients were calculated between dietary OBS components.

Cox proportional regression models were fitted with age as the underlying time variable to assess the risk of all-cause mortality, CVD death, cancer mortality, and other causes of death across OBS quartiles. Follow-up for each participant was calculated from the date the baseline questionnaire was returned to the date of death reported or the last questionnaire was received, whichever came first. We calculated hazard ratios (HRs) and 95% confidence intervals (CIs) across OBS quartiles for all-cause and cause-specific mortality, using the lowest OBS quartile (Q1) as the reference for all models. In addition, linear trend tests were performed by assigning medians to each quintile and treating it as a continuous variable.

After conducting crude analyses, we fitted different models to control for potential confounders for the effect of the OBS on mortality risk: *model 1* was adjusted for age (underlying variable) and sex; *model 2* was further adjusted for family history of CVD (dichotomous), prevalent cancer, depression, CVD, diabetes, dyslipidemia, and hypertension (all dichotomous); and *model 3* was additionally adjusted for following a special diet at baseline (dichotomous), marital status (married, single and others), MedDiet adherence (continuous), total energy intake (continuous), use of aspirin (dichotomous), and years of higher education (continuous). Furthermore, competing risk analyses based on the Fine and Gray method [40] were conducted to calculate sub-distribution hazard ratios (sHRs) and 95% CIs and eliminate the interference of competing events. As recommended by Stensrud and Hernán, no statistical test for proportional hazards was conducted [41].

We stratified our analyses by sex, age (< 60 and ≥ 60 years), and presence of chronic conditions (< 1 and ≥ 1), and we assessed effect modification between these variables and OBS quartiles by testing an interaction product-term (3 degrees of freedom) with the maximum likelihood ratio test. We additionally evaluated the specific contribution of each of the individual components of the OBS (treated as continuous variables) to the association with all-cause mortality by removing one component at a time from the total score and including the same component in the model as a covariate. Lastly, multiple sensitivity analyses were performed to test the robustness of the findings by repeating the multivariable-adjusted Cox regression models under different scenarios: excluding participants with < 2 years of follow-up before March 2017: participants deceased (*n* = 41), dropped out (*n* = 352) or answered the first follow-up questionnaire before the first 2 years of follow-up (*n* = 86); excluding participants with < 2 years of follow-up and truncating the follow-up at 10 years (*n* = 18,082); excluding participants with < 40 years at the end of the follow-up (*n* = 13,881); excluding participants with < 4 years of follow-up (*n* = 17,105); excluding participants with < 10 years of follow-up (*n* = 12,500); and re-calculating the OBS according to total vitamin C, total vitamin E, beta-carotenes, selenium, total zinc, and heme iron sex-specific quintile values (*n* = 18,561).

All analyses were conducted with Stata version 15.0 (StataCorp, College Station, TX). All *p* values are two-sided and were considered statistically significant at *p* < 0.05.

## Results

### Baseline characteristics

A total of 18,561 Spanish adults (mean [SD] age, 38.5 [12.4] years; 7,580 [40.8%] male) were included in the present analyses. During a median (interquartile range) follow-up time of 12.2 (8.3–14.9) years, 421 total deaths occurred, including 80 deaths from CVD, 215 from cancer, and 126 from other causes.

The correlations between each dietary component of the OBS are shown in Additional File 1: Table s2. Most of the coefficients revealed a low or moderate correlation between them, except for the TAC and PAC score (*r* = 0.702), the TAC and Total Vitamin C (*r* = 0.635) and the PAC score and Total Vitamin C (*r* = 0.662).

Table 1 shows the characteristics of study participants at baseline according to quartiles of OBS. Participants in the highest quartile of the OBS (> 33 points) were more likely to be women, slightly older, and had better adherence to the MedDiet. As OBS increased, participants had a higher intake of energy, carbohydrates, and fiber, but lower intake of protein, fat, and alcohol. As expected, TAC and intakes of antioxidant components, such as vitamin C, vitamin E, beta-carotene, selenium, zinc, polyphenol, and physical activity scores, increased across successive quartiles of the OBS. Regarding pro-oxidant components, the proportion of smokers and mean BMI decreased across successive quartiles of the OBS, while dietary intake of heme iron and alcohol intake score barely changed.

**Table 1:** Participants’ characteristics according to quartiles of the oxidative balance score (OBS) at baseline in the SUN cohort (*n* = 18,561)

Characteristics	Quartiles of oxidative balance score			
	Q1	Q2	Q3	Q4
N (frequency)	4976	4458	5223	3904
OBS range	5–22	23–27	28–33	34–47
Age, years	37.9 ± (12.4)	38.3 ± (12.3)	38.8 ± (12.4)	39.1 ± (12.3)
Age, median years (IQR)	35.5 (27.8–46.5)	36.3 (28.3–46.8)	37.0 (28.3–47.8)	37.5 (28.8–48.3)
Sex, men	2508 (50%)	1893 (42%)	1996 (38%)	1183 (30%)
Marital status				
Married	2513 (51%)	2275 (51%)	2754 (53%)	1941 (50%)
Singles	2189 (44%)	1937 (43%)	2199 (42%)	1708 (44%)
Others	274 (6%)	246 (6%)	270 (5%)	255 (7%)
Cumulative smoking habit, pack-years	6.7 ± (11.4)	5.3 ± (10.0)	4.4 ± (9.2)	3.0 ± (7.3)
Alcohol, g/d	7.5 ± (12.1)	7.0 ± (10.8)	6.8 ± (9.5)	5.5 ± (7.8)
Years at the university	5.1 ± (1.5)	5.1 ± (1.6)	5.1 ± (1.5)	5.0 ± (1.6)
Total energy intake, kcal/d	1949 ± (546)	2270 ± (559)	2486 ± (557)	2737 ± (530)
Carbohydrate intake, % E	40.7 ± (7.6)	42.5 ± (6.9)	43.9 ± (7.0)	46.5 ± (6.9)
Protein intake, % E	18.4 ± (3.8)	18.2 ± (3.2)	18.2 ± (3.2)	18.0 ± (3.1)
Fat intake, % E	38.2 ± (6.7)	37.2 ± (6.4)	36.0 ± (6.3)	34.1 ± (6.2)
MUFA, % E	16.2 ± (3.9)	16.0 ± (3.7)	15.5 ± (3.6)	14.8 ± (3.5)
PUFA, % E	5.3 ± (1.6)	5.2 ± (1.5)	5.1 ± (1.5)	4.9 ± (1.4)
SFA, % E	13.6 ± (3.3)	12.8 ± (2.9)	12.1 ± (2.9)	10.8 ± (2.9)
Fiber intake, g/d	17.3 ± (5.8)	23.9 ± (6.0)	30.7 ± (8.1)	42.4 ± (12.9)
^1^MedDiet adherence score, points	3.1 ± (1.5)	3.9 ± (1.6)	4.8 ± (1.6)	5.7 ± (1.5)
Oxidative balance score components				
^2^Total vitamin C, mg/d	157.8 ± (64.8)	232.8 ± (80.2)	317.6 ± (113.3)	461.7 ± (181.2)
^2^Total vitamin E, mg/d	5.3 ± (4.2)	6.9 ± (4.9)	8.3 ± (8.4)	10.5 ± (10.5)
Beta-carotene, mcg/d	2617 ± (2428)	4371 ± (3375)	6652 ± (5315)	10,450 ± (7976)
^2^Total Zinc, mg/d	12.8 ± (7.1)	16.4 ± (9.4)	19.6 ± (10.8)	24.7 ± (13.8)
Selenium, mcg/d	76.8 ± (29.1)	91.9 ± (33.1)	101.9 ± (33.5)	114.1 ± (32.3)
Heme iron, mg/d	1.9 ± (0.7)	2.1 ± (0.7)	2.1 ± (0.7)	2.1 ± (0.7)
^3^Polyphenols antioxidant content score, points	-10.6 ± (7.6)	-2.8 ± (7.2)	3.8 ± (7.3)	11.2 ± (6.8)
Flavanols, mg/d	169.7 ± (153.7)	234.0 ± (201.7)	280.6 ± (204.8)	375.6 ± (258.8)
Anthocyanins, mg/d	28.3 ± (30.2)	41.0 ± (45.0)	61.0 ± (74.3)	89.4 ± (117.2)
Flavanones, mg/d	13.1 ± (22.4)	19.9 ± (31.5)	27.4 ± (35.5)	38.7 ± (46.3)
Flavones, mg/d	8.4 ± (5.8)	11.2 ± (7.0)	13.7 ± (8.1)	16.9 ± (10.4)
Flavonols, mg/d	23.8 ± (15.0)	34.1 ± (19.2)	44.3 ± (25.5)	60.6 ± (39.2)
Isoflavonoids, mg/d	0.03 ± (0.03)	0.04 ± (0.03)	0.04 ± (0.04)	0.05 ± (0.05)
Lignans, mg/d	0.42 ± (0.28)	0.56 ± (0.33)	0.66 ± (0.36)	0.81 ± (0.42)
^4^Total antioxidant capacity, mmol Fe^+2^/100 g	5.7 ± (2.5)	7.3 ± (2.8)	8.8 ± (3.0)	11.1 ± (3.4)
BMI at baseline, kg/m^2^	24.4 ± (3.9)	23.8 ± (3.4)	23.5 ± (3.3)	22.9 ± (2.8)
^5^Alcohol intake score, points	3.6 ± (0.8)	3.6 ± (0.7)	3.6 ± (0.7)	3.7 ± (0.6)
^6^Physical activity score, points	3.0 ± (1.6)	3.5 ± (1.6)	3.8 ± (1.6)	4.4 ± (1.6)
Smoking status at baseline				
Never smoker	1664 (33%)	2038 (46%)	2714 (52%)	2486 (64%)
Current smoker	1792 (36%)	1058 (24%)	916 (18%)	315 (8%)
Former smoker	1520 (31%)	1362 (31%)	1593 (30%)	1103 (28%)

Values are means (SDs) or number of participants (percentages) unless otherwise indicated

*BMI* body mass index, *IQR* interquartile range, *MedDiet score* Mediterranean diet adherence, *MUFA* monounsaturated fatty acids, *PUFA* polyunsaturated fatty acids, *Q* quartile, *SD* standard deviation, *SFA* saturated fatty acids

^1^Score proposed by Trichopoulou et al.[39]

^2^Total accounts for intakes from both dietary and supplemental sources

^3^Score proposed by Pounis et al.[29]

^4^Calculated from an Antioxidant Food Database base proposed by Carlsen et al.[30]

^5^Score according to criteria of the OBS

^6^Score proposed by Alvarez-Alvarez et al.[38]

### OBS and mortality risk

Associations of the OBS with all-cause, CVD, cancer, and other-cause mortality are summarized in Table 2. Crude and fully adjusted models showed the risk of all-cause and cause-specific mortality was reduced linearly across successive quartiles of the OBS (*p* < 0.050 for trend). For other-cause mortality, this trend was non-significant (*p* = 0.054 for trend). In the fully adjusted model (Model 3), a higher OBS was associated with statistically significant lower all-cause, CVD, and cancer mortality risk. Among participants in the highest OBS quartile, the mortality relative risk reduction was 65% (HR 0.35 [95% CI 0.22–0.54]) for all-cause mortality, 82% (HR 0.18 [95% CI 0.06–0.51]) for CVD mortality, and 65% (HR 0.35 [95% CI 0.19–0.65]) for cancer mortality, as compared with participants in the lowest OBS quartile. For other-cause mortality, a non-significant 55% relative risk reduction (HR 0.45 [95% CI 0.20–1.02]) was observed when comparing the highest *vs.* the lowest quartile of the OBS.

**Table 2:** Associations of the oxidative balance Score (OBS) with all-cause, cancer, cardiovascular, and other-cause mortality in the SUN cohort (*n* = 18,561)

	Quartiles of oxidative balance score				
	Q1	Q2	Q3	Q4	p for trend
*n* (frequency)	4976	4458	5223	3904	
OBS range	5–22	23–27	28–33	34–47	
Person-years	57,291	52,184	60,728	43,827	
All-cause mortality					
Deaths	132	124	122	43	
Mortality rate/10000 person-years	23.04	23.76	20.09	9.81	
Crude model	1 (Ref.)	1.03 (0.81–1.31)	0.86 (0.67–1.10)	0.42 (0.29–0.59)	< 0.001
Model 1	1 (Ref.)	1.13 (0.88–1.45)	0.85 (0.66–1.09)	0.43 (0.30–0.62)	< 0.001
Model 2	1 (Ref.)	1.18 (0.92–1.52)	0.88 (0.68–1.13)	0.46 (0.32–0.65)	< 0.001
Model 3	1 (Ref.)	1.07 (0.82–1.41)	0.74 (0.54–1.02)	0.35 (0.22–0.54)	< 0.001
CVD mortality					
Deaths	31	23	18	8	
Mortality rate/10000 person-years	5.41	4.41	2.96	1.83	
Crude model	1 (Ref.)	0.81 (0.47–1.39)	0.54 (0.30–0.97)	0.33 (0.15–0.71)	0.001
Model 1	1 (Ref.)	0.93 (0.54–1.62)	0.60 (0.33–1.10)	0.38 (0.17–0.84)	0.003
Model 2	1 (Ref.)	1.03 (0.58–1.82)	0.62 (0.34–1.14)	0.41 (0.18–0.91)	0.013
Model 3	1 (Ref.)	0.77 (0.42–1.41)	0.36 (0.17–0.77)	0.18 (0.06–0.51)	0.001
Cancer mortality					
Deaths	59	66	69	21	
Mortality rate/10000 person-years	10.30	12.65	11.36	4.79	
Crude model	1 (Ref.)	1.22 (0.86–1.73)	1.09 (0.77–1.54)	0.45 (0.27–0.74)	0.009
Model 1	1 (Ref.)	1.30 (0.91–1.86)	1.06 (0.74–1.51)	0.44 (0.27–0.74)	0.009
Model 2	1 (Ref.)	1.31 (0.92–1.88)	1.10 (0.77–1.57)	0.46 (0.28–0.77)	0.014
Model 3	1 (Ref.)	1.18 (0.80–1.73)	0.91 (0.59–1.40)	0.35 (0.19–0.65)	0.002
Other-cause mortality					
Deaths	42	35	35	14	
Mortality rate/10000 person-years	7.33	6.71	5.76	3.19	
Crude model	1 (Ref.)	0.92 (0.59–1.44)	0.78 (0.50–1.22)	0.43 (0.23–0.79)	0.007
Model 1	1 (Ref.)	1.03 (0.65–1.63)	0.71 (0.45–1.13)	0.47 (0.25–0.89)	0.013
Model 2	1 (Ref.)	1.06 (0.67–1.69)	0.71 (0.44–1.13)	0.48 (0.25–0.91)	0.012
Model 3	1 (Ref.)	1.06 (0.64–1.76)	0.71 (0.40–1.27)	0.45 (0.20–1.02)	0.054

Hazard ratios (HR) and 95% confidence intervals (CI)

*CVD* cardiovascular disease, *OBS* oxidative balance score, *Q* quartile, *ref.* reference

Model 1: adjusted for age (underlying variable) and sex (dichotomous), and stratified by deciles of age and recruitment period (6 categories)

Model 2: additionally adjusted for family history of cardiovascular diseases (dichotomous), prevalent cancer (dichotomous), prevalent depression (dichotomous), prevalent cardiovascular disease* (dichotomous), prevalent diabetes (dichotomous), prevalent dyslipidaemia (dichotomous), prevalent hypertension (dichotomous)

Model 3: additionally adjusted for following special diet at baseline (dichotomous), marital status (married, single and others), Mediterranean diet adherence (continuous), total energy intake (continuous), use of aspirin (dichotomous), and years of higher education (continuous)

*Prevalent cardiovascular disease was considered as having at least one of the following events before entering the cohort: aneurysm, angina pectoris, atrial fibrillation, cardiac insufficiency, coronary bypass, deep vein thrombosis, intermittent claudication, myocardial infarction, pulmonary embolism, stroke, or tachycardia

In competing risk analyses (Additional File 1: Table s3), the association between the OBS and cancer and CVD mortality remained statistically significant, although the magnitude of the risk reduction was attenuated when comparing the highest OBS relative to the lowest OBS quartile.

We assessed the association of the dietary OBS components (vitamins C and E, β-carotenes, selenium, zinc, heme iron, polyphenols, and total antioxidant capacity) separately from the lifestyle OBS components (body mass index, alcohol, smoking, and physical activity) with mortality risk (Additional File 1: Table s4). Inverse associations were attenuated with respect to the main analyses for both the dietary and the lifestyle OBS components, however, estimated associations for the dietary OBS components lost significance (except for cancer mortality). We additionally explored the association of each item of the score with all-cause and cause-specific mortality risk (Additional File 1: Table s5). As expected, unhealthy lifestyles, such as alcohol, physical inactivity, and smoking, resulted to be significantly associated with mortality risk, particularly extremes of behaviors. No associations were found between isolate dietary components and mortality outcomes.

### Stratified analyses

The results of the crude and multivariable-adjusted associations of the OBS with all-cause mortality risk stratified by sex, age, and presence of chronic diseases are shown in Table 3. The magnitude of the inverse association between OBS and all-cause mortality was greater for women, older participants (≥ 60 years), and those without any chronic condition, but we did not observe any statistically significant interaction.

**Table 3:** Associations of the oxidative balance score (OBS) with all-cause, cancer, cardiovascular, and other-cause mortality stratified by sex, age and prevalent chronic conditions in the SUN cohort (*n* = 18,561)

		Quartiles of oxidative balance score											
		Q1	Q2	Q3	Q4	*p* for trend		Q1	Q2	Q3	Q4	*p* for trend	*p* for interaction
***Sex***		**Men**						**Women**					
*n* (frequency)		2169	1861	1782	1768			2918	2669	2673	2721		
OBS range		5–21	22–26	27–31	32–47			8–23	24–28	29–33	34–46		
Person-years		25,083	22,053	21,121	20,595			33,434	30,649	30,906	30,189		
**All-cause mortality**													
Deaths		95	91	79	48			27	35	27	19		
Mortality rate/10000 person-years		37.87	41.26	37.40	23.31			8.08	11.42	8.74	6.29		
Crude model		1 (Ref.)	1.09 (0.82–1.45)	0.97 (0.72–1.31)	0.60 (0.42–0.85)	0.006		1 (Ref.)	1.40 (0.85–2.31)	1.07 (0.63–1.82)	0.76 (0.42–1.37)	0.283	0.816
Multivariable model		1 (Ref.)	1.18 (0.85–1.62)	0.82 (0.56–1.19)	0.45 (0.28–0.72)	0.001		1 (Ref.)	1.07 (0.61–1.87)	0.67 (0.36–1.28)	0.34 (0.15–0.77)	0.005	0.070
***Age***		** < 60 Years**						** ≥ 60 Years**					
n (frequency)		4710	4232	4927	3683			266	281	241	221		
OBS range		5–22	23–27	28–33	34–47			10–22	23–28	29–33	34–45		
Person-years		54,437	49,822	57,549	41,332			2854	2928	2612	2495		
**All-cause mortality**													
Deaths		73	69	63	24			59	68	46	19		
Mortality rate/10000 person-years		13.41	13.85	10.95	5.81			206.7	232.2	176.1	76.15		
Crude model		1 (Ref.)	1.03 (0.74–1.43)	0.81 (0.58–1.13)	0.42 (0.27–0.67)	< 0.001		1 (Ref.)	1.14 (0.80–1.61)	0.79 (0.53–1.16)	0.34 (0.20–0.58)	< 0.001	0.879
Multivariable model		1 (Ref.)	1.06 (0.74–1.52)	0.77 (0.51–1.17)	0.41 (0.23–0.73)	0.004		1 (Ref.)	1.08 (0.71–1.64)	0.65 (0.39–1.07)	0.28 (0.14–0.57)	< 0.001	0.846
***Chronic Diseases***		** < 1 Chronic disease**						** ≥ 1 Chronic diseases**					
n (frequency)		3448	3200	3665	2850			1528	1258	1313	1299		
OBS range		6–22	23–27	28–33	34–47			5–22	23–27	28–32	33–46		
Person-years		40,215	37,881	42,943	32,174			17,076	14,303	15,009	14,428		
**All-cause mortality**													
Deaths		46	40	31	13			86	84	80	41		
Mortality rate/10000 person-years		11.44	10.56	7.22	4.04			50.36	58.73	53.3	28.42		
Crude model		1 (Ref.)	0.92 (0.60–1.40)	0.62 (0.40–0.98)	0.34 (0.19–0.64)	< 0.001		1 (Ref.)	1.16 (0.86–1.57)	1.04 (0.77–1.41)	0.55 (0.38–0.80)	0.004	0.275
Multivariable model		1 (Ref.)	0.91 (0.57–1.46)	0.62 (0.35–1.08)	0.28 (0.13–0.63)	< 0.001		1 (Ref.)	1.14 (0.82–1.60)	0.83 (0.57–1.21)	0.41 (0.25–0.68)	0.001	0.393

Hazard ratios (HR) and 95% confidence intervals (CI)

Multivariable model: adjusted for age (underlying variable), family history of cardiovascular diseases (dichotomous), following special diet at baseline (dichotomous), marital status (married, single and others). Mediterranean adherence (continuous), prevalent cancer (dichotomous), prevalent depression (dichotomous), prevalent cardiovascular disease* (dichotomous), prevalent diabetes (dichotomous), prevalent dyslipidaemia (dichotomous), prevalent hypertension (dichotomous), sex (dichotomous), total energy intake (continuous), use of aspirin (dichotomous), years of higher education (continuous), and stratified by deciles of age and recruitment period (6 categories)

*CVD* cardiovascular disease, *OBS* oxidative balance score, *Q* quartile, *ref.* reference

*Prevalent cardiovascular disease was considered as having at least one of the following events before entering the cohort: aneurysm, angina pectoris, atrial fibrillation, cardiac insufficiency, coronary bypass, deep vein thrombosis, intermittent claudication, myocardial infarction, pulmonary embolism, stroke, or tachycardia

### Contribution of the individual components of the OBS

**Fig. 2:**
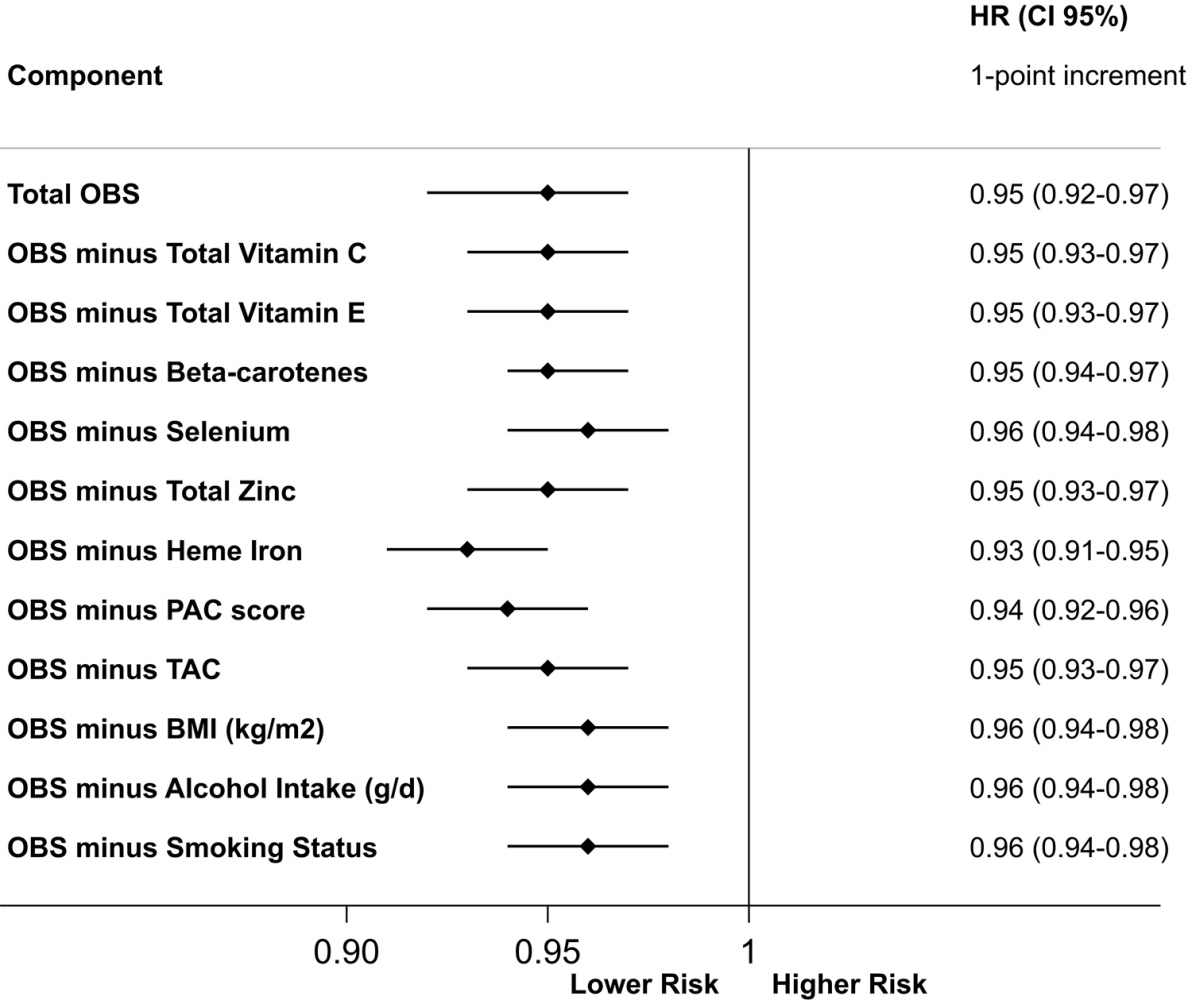
All-cause mortality hazard ratios (HRs) and 95% confidence intervals (CIs) per 1-point increment associated with the oxidative balance score (OBS) and after alternate subtraction of each of its dietary components. All models were adjusted for age (underlying variable), family history of cardiovascular diseases (dichotomous), following special diet at baseline (dichotomous), marital status (married, single and others). Mediterranean adherence (continuous), prevalent cancer (dichotomous), prevalent depression (dichotomous), prevalent cardiovascular disease* (dichotomous), prevalent diabetes (dichotomous), prevalent dyslipidemia (dichotomous), prevalent hypertension (dichotomous), sex (dichotomous), total energy intake (continuous), use of aspirin (dichotomous), years of higher education (continuous), corresponding subtracted component, and stratified by deciles of age and recruitment period (6 categories). *Prevalent cardiovascular disease was considered as having at least one of the following events before entering the cohort: aneurysm, angina pectoris, atrial fibrillation, cardiac insufficiency, coronary bypass, deep vein thrombosis, intermittent claudication, myocardial infarction, pulmonary embolism, stroke, or tachycardia

After removing each of the OBS components one at a time and adjusting for the removed component using it as a covariate, the differences in mortality risk estimates remained significant and barely changed (a maximum difference of 2% with respect to the primary OBS analysis) (Fig. 2).

### Sensitivity analyses

Consistent with the primary analyses, all point estimates showed an inverse association between the OBS and risk of all-cause and cause-specific mortality for all the sensitivity analyses. Results remained similar in most scenarios, and in some models, the inverse association became stronger, suggesting a robust association between the OBS and mortality risk (Additional File 1: Figure s1). Noteworthy, the OBS association with other-cause mortality became significant in most of the proposed scenarios.

## Discussion

We investigated the association of overall oxidative balance with all-cause, CVD, cancer, and other-cause mortality risk among nearly 20,000 middle-aged Spanish adults in a Mediterranean cohort. We used a novel holistic score based on 12 a priori selected dietary and non-dietary lifestyle pro- and antioxidants exposures to represent the overall oxidative balance status of an individual in a comprehensive manner. Our results suggested a statistically significant strong inverse association between the OBS and all-cause and cause-specific mortality. In addition, our stratified analyses suggested that women, older participants, and those without any chronic conditions may experience lower mortality risk when achieving a better antioxidant balance status, although no significant interaction effect was observed.

Oxidative stress is a well-studied topic in research, with the theory of aging having been proposed decades ago [41]. Several clinical trials and cohort studies have assessed the possible protective role of antioxidants in the prevention of cardiovascular disease, cancer, or premature death, among others, observing inconsistent findings [42–44]. In our study, we did not find any association between isolate dietary components and mortality risk. Possible reasons of these inconclusive results may rely on the synergistic and interactive effect among nutrients in the food matrix, which exert a greater effect than the corresponding action of the individual antioxidants. Also, antioxidants’ health benefits depend on both intake levels and bioavailability, and the latter is affected by food processing. Lastly, environmental conditions, production, harvest, or cultivation techniques might as well impact the antioxidant food content [45–47]. Epidemiological studies have since sought to demonstrate how antioxidant exposures may positively impact health outcomes and reduce premature mortality. Although most efforts in the past decades have focused on examining individual antioxidant dietary [48, 49] or lifestyle exposures [50–52], there has been a recent shift toward a more comprehensive approach to assess the overall oxidative balance by combining different exposures in a single score, namely the OBS [13]. Despite the considerable heterogeneity in the definitions of the OBS that have emerged in the last years, more robust and consistent evidence has been observed for the inverse association between recently developed OBSs and health outcomes, including certain types of cancer, CVD risk factors, and biomarkers [13]. However, only two previous studies have examined the association between an OBS (comprising both dietary and lifestyle exposure) and mortality risk before the present analyses. These studies showed similar findings to our study [15, 16]. In 2015, Kong et al. [15] published a population-based prospective cohort study aiming to examine reasons for variations in stroke incidence and mortality. The study included 21,031 black and white individuals aged 45 years or older with oversampling of persons from the “stroke belt”. These authors reported an inverse association between higher OBS score (14 a priori components: 10 dietary and 4 lifestyle factors), and all-cause, cancer, and non-cancer mortality, with a significantly lower mortality risk of 30%, 50%, and 33%, respectively. Associations for cardiac and heart failure mortality, however, were not significant. As secondary analyses, the contribution of each component of the OBS showed modest differences in risk estimates for the majority of the components, consistent with our findings. This OBS differs from ours in the inclusion of other carotenoids (i.e., lycopene, α-carotene, lutein, β-cryptoxanthin), polyunsaturated fatty acids, and regular use of aspirin and NSAIDs, and the exclusion of zinc, physical activity and BMI. In another study conducted in the Iowa Women’s Health study, Mao et al. [16] reported similar associations between women with higher OBS score (15 a priori factors: 11 dietary and 4 lifestyle factors) and all-cause, CVD, and cancer mortality with a significantly lower mortality risk of 34%, 39%, and 29%, respectively. This OBS included the same non-dietary factors as our OBS, and included other carotenoids (i.e., α-carotene, lutein, zeaxanthin, lycopene) and polyunsaturated fatty acids as well as saturated fats. These authors [16] concluded that associations with mortality seemed to be mainly driven by lifestyle components, observing the strongest association for the OBS composed of only lifestyle components. However, in our study, we found that the potential protective effect of an only-lifestyle OBS was smaller than the combination of both dietary and lifestyle exposures, suggesting a potential synergism between the components of the score that are not captured by individual assessments. Although little is known about the mechanism of interactive or synergistic actions, it is reasonable to assume that a combination of antioxidant systems (dietary and lifestyle) would have greater beneficial effects than a single factor alone or even than the sum of isolated factors. In other words, the total might exert a greater effect than the sum of its parts. Given that several factors may affect multiple pathways, lifestyle and dietary factors may synergistically work together to protect the body against free radical damage [53]. Finally, it is well known that the relationship between oxidative stress and inflammation, and both processes are connected with NCD development. In a previous analysis, we observed an association between the pro-inflammatory capacity of diet, measured with the dietary inflammatory index and mortality risk in the SUN and PREDIMED studies [54–56]. In the SUN cohort, the correlation between this dietary inflammatory index and our OBS was moderately strong (*r* = − 0.810, *p* < 0.001). It would be interesting to explore in future the effect of a new score combining both the inflammatory and oxidative effect of diet and other lifestyles on mortality risk.

Our study may help better understand the beneficial effect of a combined set of dietary and lifestyle factors on the prevention of mortality centered on their potential to reduce oxidative stress. Based on our results, the combination of non-smoking, low consumption or abstinence of alcohol, regular physical activity, and maintaining a normal BMI has a strong antioxidant effect, which could be helpful toward the prevention of premature mortality. Moreover, following a dietary pattern rich in antioxidant compounds may further contribute to this preventive effect. In this regard, identification of individuals with an adequate oxidant balance provides a novel approach to design multidimensional interventions aimed at improving dietary patterns accompanied by healthy lifestyle behavior changes. A shift from a unidimensional to a more multidimensional approach with dietary and lifestyle interventions may be warranted in the current nutritional epidemiology.

Certain limitations of our study should be acknowledged. First, information used to construct the OBS was collected at baseline, and participants may have modified their dietary and lifestyle exposures throughout follow-up. However, we were not able to update the OBS because a new FFQ was collected only after 10 years of follow-up, other lifestyle factors were not available in this follow-up questionnaire, and the number of participants with information available at this time was limited. Second, our OBS may not capture certain antioxidant factors, such as total fat, certain PUFAS (ω-6 and ω-3), saturated fatty acids, vitamin D, folate, calcium, and fiber; however, diverse discrepancies arose about the inclusion of these factors in OBSs [13]. Information about carotenoids intake, beyond β-carotene was not available in our database, and it will be interesting to replicate our analyses with this additional information. On the other hand, diverse discrepancies arose about the inclusion of these factors in OBSs [13]. Regarding fat or fatty acids, polyunsaturated fatty acids (PUFAs) have been included as pro-oxidant component of previous OBS [13]. However, there is still controversy about the effect of omega-6 fatty acids and their lipid mediators, their mechanism of action and interaction with omega-3 fatty acids [58]. Third, our equally weighted OBS scoring system may be questionable; yet our findings are in line with those studies that used different weight OBS scoring systems [57]. Fourth, we did not collect information of biomarkers regarding patients’ oxidative status. However, two recently published articles have assessed the possible relation of OBSs and these biomarkers in adult populations, finding an inverse association between OBSs and C-reactive protein [58], and plasma F_2_-isoprostanes [59]. Fifth, although we adjusted for aspirin use and other factors, certain endogenous and other exogenous factors such as medications may affect the oxidative balance of an individual. Sixth, although the semi-quantitative FFQ used in this study has been repeatedly validated with good reproducibility and relative validity [24–26], self-reported questionnaires may be prone to some degree of measurement error. Seventh, our analyses supported a strong inverse association between the OBS and mortality risk in a fairly young cohort. These findings support that an antioxidant dietary and lifestyle may reduce premature mortality risk. However, replication of our findings in larger and older cohorts with higher mortality and cause-specific mortality cases should be warranted. Additionally, residual confounding may exist, yet we adjusted for multiple confounders to reduce this possibility. Lastly, the characteristics of the SUN cohort, a middle-aged university graduates with relatively low prevalence of risk factors for chronic diseases, may compromise the generalization of the results. However, this restriction to university graduates may represent an advantage because these participants’ characteristics confer robust internal validity to our results and greater reliability on the self-reported information in addition to reducing the potential confounding related to socioeconomic status and educational level. Despite these limitations, the major strengths of the study rely on the inclusion of components that have not been previously covered by any of the proposed OBSs (such as PAC score and TAC), the relatively large sample of the study, the long average follow-up, the high retention rate, the verification of mortality cases by medical records or consultation of the National Death Index, the adjustment for many potential confounders, and the robustness of the results found in the several sensitivity analyses performed.

## Conclusions

Our findings suggest that greater antioxidant balance status is associated to lower premature mortality, including CVD and cancer-related mortality. Individuals exposed to both antioxidant dietary and lifestyle exposures may potentially experience the lowest all-cause and cause-specific mortality risk. Moreover, the results of the present study support the utility of our OBS to capture potential correlations and synergies between the antioxidant components that single assessments may not capture. Efforts to prevent premature mortality should be focused on recommending dietary patterns rich in antioxidant compounds together with healthy lifestyle behaviors.

## Additional information

The findings of the study have been presented as a virtual oral presentation at the American Society for Nutrition meeting, NUTRITION 2021 LIVE ONLINE, and the presentation has been recognized as finalist for the American Society for Nutrition’s Emerging Leaders in Nutrition Science Abstract Recognition Award Program.

## Supplementary Information

Below is the link to the electronic supplementary material.

Supplementary file1 (DOCX 237 KB)

## Data Availability

Data described in the manuscript, code book, and analytic code will be made available upon request pending application and approval.
