# Supplementary material for: Association between an oxidative balance score and mortality: a prospective analysis in the SUN cohort
Source: Eur J Nutr. 2023 Feb 12;62(4):1667–80. doi: 10.1007/s00394-023-03099-8 (PMC10195723; doi:10.1007/s00394-023-03099-8)
Supplement: Supplementary file 1 — Supplementary file1 (DOCX 237 KB) [file 394_2023_3099_MOESM1_ESM.docx]

**SUPPLEMENTARY INFORMATION**

**Association between an oxidative balance score and mortality: a prospective analysis in the SUN cohort**

Irene Talavera-Rodríguez*, Cesar I Fernandez-Lazaro*, Ángela Hernández-Ruiz, María Soledad Hershey, Cristina Gallarregui, Mercedes Sotos-Prieto, Carmen de la Fuente-Arrillaga, Miguel Ángel Martínez-González, Miguel Ruiz-Canela
*both first and second authors contributed equally to this work

**Supplemental Table s1:** Criteria for scoring the oxidative balance score (OBS).

**Supplemental Table s2:** Pearson product-moment correlation coefficients between the dietary components of the OBS.

**Supplemental Table s3:** Competing risk analysis. Proportional subdistribution hazards models for the associations of the oxidative balance score (OBS) with cancer, cardiovascular, and other-cause mortality in the SUN cohort (n = 18,561).

**Supplemental Table s4:** Associations of the oxidative balance score (OBS) dietary and lifestyle components with all-cause, cancer, cardiovascular, other-cause mortality in the SUN cohort (n = 18,561). Hazard ratios (HR) and 95% confidence intervals (CI).

**Supplemental Table s5.** Associations of the items included in the oxidative balance score (OBS) with all-cause, cancer, cardiovascular, and other-cause mortality in the SUN cohort (n = 18,561). Hazard ratios (HR) and 95% confidence intervals (CI).

**Supplemental Figure s1.** Sensitivity analyses. Hazard Ratio (HR) and 95% Confidence Interval (CI) for the associations of the oxidative balance score (OBS) with all-cause, cancer, cardiovascular, and other-cause mortality under different assumptions: A) excluding participants with < 2years of follow-up (n= 18,082); B) truncating the participants’ follow-up at 10 years (n= 18,561); C) excluding participants with < 2 years of follow-up and truncating the follow-up at 10 years (n=18,082); D) excluding participants with < 40 years at the end of the follow-up (n=13,881); E) excluding participants with < 4 years of follow-up (n= 17,105); F) excluding participants with < 10 years of follow-up (n= 12,500); G) re-calculating the OBS according to total vitamin C, total vitamin E, beta-carotenes, selenium, total zinc, and heme iron sex-specific quintile values (n=18,561), in the SUN cohort (1999–2018).

**TABLES**

| **Supplemental Table 1.** Criteria for scoring the oxidative balance score (OBS). | | | | | |
| --- | --- | --- | --- | --- | --- |
|  | **0 points** | **1 point** | **2 points** | **3 points** | **4 points** |
| ^1^Total Vitamin C (mg/d) | Q1 | Q2 | Q3 | Q4 | Q5 |
| ^1^Total Vitamin E (mg/d) | Q1 | Q2 | Q3 | Q4 | Q5 |
| Dietary Beta-carotenes (mcg/d) | Q1 | Q2 | Q3 | Q4 | Q5 |
| Dietary Selenium (mcg/d) | Q1 | Q2 | Q3 | Q4 | Q5 |
| ^1^Total Zinc (mg/d) | Q1 | Q2 | Q3 | Q4 | Q5 |
| Dietary Heme Iron (mg/d) | Q5 | Q4 | Q3 | Q2 | Q1 |
| ^2^Dietary PAC score | Q1 | Q2 | Q3 | Q4 | Q5 |
| Dietary TAC (mmol Fe^+2^/100g) | Q1 | Q2 | Q3 | Q4 | Q5 |
| BMI (kg/m^2^) | ≥35 | <35 & ≥30 | <30 & ≥27 | <27 & ≥25 | <25 |
| Alcohol Intake (g/d) | ♂ >75 | ≤75 & >50 | ≤50 & >20 | ≤20 & >10 | ≤10 |
|  | ♀ >50 | ≤50 & >25 | ≤25 & >15 | ≤15 & >5 | ≤5 |
|  | **0 points** | **2 points** | | **4 points** | |
| Smoking status | Current | Former | | Never | |
| ^3^PA score | 0-2 | 3-5 | | 6-8 | |

**Abbreviations**: BMI, body mass index; PAC, polyphenols antioxidant content score; TAC, total antioxidant capacity; PA, physical activity; Q, quartiles.
^1^Total accounts for intakes from both dietary and supplemental sources.

^2^Score proposed by Pounis et al. (29)

^3^Score proposed by Alvarez-Alvarez et al. (38)

| **Supplemental Table s2.** Pearson product-moment correlation coefficients between the dietary components of the OBS. | | | | | | | |
| --- | --- | --- | --- | --- | --- | --- | --- |
| **Components** | **Dietary TAC** | **Total Vitamin C** | **Total Vitamin E** | **Dietary Beta-carotenes** | **Dietary Selenium** | **Total Zinc** | **Dietary Heme Iron** |
| **^1^Total Vitamin C** (mg/d) | 0.635 | -- |  |  |  |  |  |
| **^1^Total Vitamin E** (mg/d) | 0.116 | 0.162 | -- |  |  |  |  |
| **Dietary Beta-carotenes** (mcg**/**d) | 0.288 | 0.518 | 0.081 | -- |  |  |  |
| **Dietary Selenium** (mcg/d) | 0.269 | 0.209 | 0.125 | 0.134 | -- |  |  |
| **^1^Total Zinc** (mg/d) | 0.238 | 0.304 | 0.097 | 0.201 | 0.246 | -- |  |
| **^2^Dietary PAC score** | 0.702 | 0.662 | 0.184 | 0.414 | 0.283 | 0.247 | -- |
| **Dietary Heme Iron** (mg/d) | 0.122 | 0.084 | 0.111 | 0.019 | 0.521 | 0.214 | 0.154 |

**Abbreviations**: d, day; mcg, micrograms; mg milligrams; OBS, oxidative balance score; PAC, polyphenol antioxidant content.

^1^Total accounts for intakes from both dietary and supplemental sources.

^2^Score proposed by Pounis et al. (29)

| **Supplemental Table s3**. Competing risk analyses. Proportional subdistribution hazards models for the associations of the oxidative balance score (OBS) with cancer, cardiovascular, and other-cause mortality in the SUN cohort (n = 18,561). | | | | | |
| --- | --- | --- | --- | --- | --- |
|  | **Quartiles of Oxidative Balance Score** | | | | |
|  | Q1 | Q2 | Q3 | Q4 | p for trend |
| n (frequency) | 4976 | 4458 | 5223 | 3904 |  |
| OBS range | 5-22 | 23-27 | 28-33 | 34-47 |  |
| Person-years | 57291 | 52183 | 60727 | 43827 |  |
| **CVD mortality** |  |  |  |  |  |
| Deaths | 31 | 23 | 18 | 8 |  |
| Mortality rate/10000 person-years | 5.41 | 4.40 | 2.96 | 1.82 |  |
| Crude model | 1 (Ref.) | 0.81 (0.47-1.39) | 0.54 (0.30-0.97) | 0.33 (0.15-0.72) | 0.001 |
| Multivariable model | 1 (Ref.) | 0.80 (0.43-1.46) | 0.39 (0.17-0.86) | 0.27 (0.10-0.69) | 0.003 |
| **Cancer mortality** |  |  |  |  |  |
| Deaths | 59 | 66 | 69 | 21 |  |
| Mortality rate/10000 person-years | 10.30 | 12.65 | 11.36 | 4.79 |  |
| Crude model | 1 (Ref.) | 1.22 (0.86-1.74) | 1.09 (0.77-1.54) | 0.45 (0.28-0.75) | 0.003 |
| Multivariable model | 1 (Ref.) | 1.13 (0.77-1.67) | 0.89 (0.59-1.35) | 0.36 (0.19-0.68) | 0.002 |
| **Other-cause mortality** |  |  |  |  |  |
| Deaths | 42 | 35 | 35 | 14 |  |
| Mortality rate/10000 person-years | 7.33 | 6.71 | 5.76 | 3.19 |  |
| Crude model | 1 (Ref.) | 0.92 (0.59-1.44) | 0.78 (0.50-1.22) | 0.43 (0.24-0.80) | 0.005 |
| Multivariable model | 1 (Ref.) | 1.07 (0.65-1.78) | 0.84 (0.49-1.44) | 0.55 (0.25-1.20) | 0.124 |

**Abbreviations**: CVD, cardiovascular disease; OBS, oxidative balance score; Q, quartile; ref., reference.

**Multivariable model:** adjusted for age (underlying variable), family history of cardiovascular diseases (dichotomous), following special diet at baseline (dichotomous), marital status (married, single and others). Mediterranean adherence (continuous), prevalent cancer (dichotomous), prevalent depression (dichotomous), prevalent cardiovascular disease* (dichotomous), prevalent diabetes (dichotomous), prevalent dyslipidaemia (dichotomous), prevalent hypertension (dichotomous), sex (dichotomous), total energy intake (continuous), use of aspirin (dichotomous), years of higher education (continuous), and stratified by deciles of age and recruitment period (6 categories).

*Prevalent cardiovascular disease was considered as having at least one of the following events before entering the cohort: aneurysm, angina pectoris, atrial fibrillation, cardiac insufficiency, coronary bypass, deep vein thrombosis, intermittent claudication, myocardial infarction, pulmonary embolism, stroke, or tachycardia.

| **Supplemental Table s4**. Associations of the oxidative balance score (OBS) dietary and lifestyle components with all-cause, cancer, cardiovascular, other-cause mortality in the SUN cohort (n = 18,561). Hazard ratios (HR) and 95% confidence intervals (CI). | | | | | | | | | | | | | | | |
| --- | --- | --- | --- | --- | --- | --- | --- | --- | --- | --- | --- | --- | --- | --- | --- |
|  | | **Quartiles of Oxidative Balance Score** | | | | | | | | | | | | | |
|  |  | **Dietary OBS** | | | | |  | | **Lifestyle OBS** | | | | | | |
|  |  | Q1 | Q2 | Q3 | Q4 | p for trend | |  | | Q1 | Q2 | Q3 | Q4 | p for trend |  |
| n (frequency) |  | 5019 | 4755 | 4688 | 4099 |  | |  | | 6989 | 5118 | 5185 | 1269 |  |  |
| OBS range |  | 0-11 | 12-16 | 17-21 | 22-32 |  | |  | | 1-10 | 11-12 | 13-14 | 15-16 |  |  |
| Person-years |  | 58111 | 55208 | 54788 | 45923 |  | |  | | 80815 | 58983 | 59784 | 14448 |  |  |
| **All-cause mortality** |  |  |  |  |  |  | |  | |  |  |  |  |  |  |
| Deaths |  | 108 | 115 | 126 | 72 |  | |  | | 250 | 111 | 48 | 12 |  |  |
| Mortality rate/10000 person-years |  | 18.59 | 20.83 | 23.00 | 15.68 |  | |  | | 30.93 | 18.82 | 8.03 | 8.31 |  |  |
| Crude model |  | 1 (Ref.) | 1.13 (0.87-1.46) | 1.23 (0.95-1.59) | 0.84 (0.62-1.13) | 0.594 | |  | | 1 (Ref.) | 0.70 (0.56-0.87) | 0.41 (0.30-0.57) | 0.53 (0.29-0.95) | <0.001 |  |
| Multivariable model |  | 1 (Ref.) | 1.18 (0.88-1.60) | 1.07 (0.75-1.52) | 0.68 (0.44-1.07) | 0.148 | |  | | 1 (Ref.) | 0.78 (0.61-0.99) | 0.47 (0.33-0.65) | 0.66 (0.36-1.19) | <0.001 |  |
| **CVD mortality** |  |  |  |  |  |  | |  | |  |  |  |  |  |  |
| Deaths |  | 25 | 24 | 15 | 16 |  | |  | | 60 | 15 | 5 | - |  |  |
| Mortality rate/10000 person-years |  | 4.30 | 4.35 | 2.74 | 3.48 |  | |  | | 7.42 | 2.54 | 0.84 | - |  |  |
| Crude model |  | 1 (Ref.) | 0.81 (0.47-1.39) | 0.54 (0.30-0.97) | 0.33 (0.15-0.71) | 0.001 | |  | | 1 (Ref.) | 0.34 (0.19-0.59) | 0.11 (0.04-0.27) | **-**^1^ | <0.001 |  |
| Multivariable model |  | 1 (Ref.) | 0.95 (0.48-1.85) | 0.42 (0.17-1.01) | 0.39 (0.14-1.11) | 0.042 | |  | | 1 (Ref.) | 0.42 (0.22-0.80) | 0.20 (0.07-0.54) | **-**^2^ | <0.001 |  |
| **Cancer mortality** |  |  |  |  |  |  | |  | |  |  |  |  |  |  |
| Deaths |  | 47 | 61 | 71 | 36 |  | |  | | 123 | 60 | 25 | 7 |  |  |
| Mortality rate/10000 person-years |  | 8.09 | 11.05 | 12.96 | 7.84 |  | |  | | 15.22 | 10.17 | 4.18 | 4.84 |  |  |
| Crude model |  | 1 (Ref.) | 1.37 (0.94-2.00) | 1.59 (1.10-2.30) | 0.96 (0.62-1.48) | 0.641 | |  | | 1 (Ref.) | 0.65 (0.48-0.89) | 0.27 (0.17-0.41) | 0.31 (0.14-0.65) | <0.001 |  |
| Multivariable model |  | 1 (Ref.) | 1.32 (0.86-2.02) | 1.29 (0.80-2.10) | 0.72 (0.39-1.34) | 0.441 | |  | | 1 (Ref.) | 0.86 (0.62-1.20) | 0.49 (0.31-0.78) | 0.73 (0.33-1.61) | 0.008 |  |
| **Other-cause mortality** |  |  |  |  |  |  | |  | |  |  |  |  |  |  |
| Deaths |  | 36 | 30 | 40 | 20 |  | |  | | 67 | 36 | 18 | 5 |  |  |
| Mortality rate/10000 person-years |  | 6.20 | 5.43 | 7.30 | 4.36 |  | |  | | 8.29 | 6.10 | 3.01 | 3.46 |  |  |
| Crude model |  | 1 (Ref.) | 0.88 (0.54-1.43) | 1.17 (0.74-1.83) | 0.70 (0.41-1.22) | 0.513 | |  | | 1 (Ref.) | 0.72 (0.48-1.08) | 0.35 (0.21-0.60) | 0.40 (0.16-1.00) | <0.001 |  |
| Multivariable model |  | 1 (Ref.) | 1.01 (0.57-1.79) | 1.17 (0.61-2.23) | 0.69 (0.30-1.63) | 0.636 | |  | | 1 (Ref.) | 0.92 (0.59-1.44) | 0.58 (0.32-1.06) | 1.03 (0.39-2.69) | 0.212 |  |

^1^There was not any CVD death in Q4. We merged Q3+Q4 resulting in three categories (Q1, Q2, and Q3+Q4) with HR (95% CI) of 0.71 (0.42-1.20) for extreme categories.

^2^There was not any CVD death in Q4. We merged Q3+Q4 resulting in three categories (Q1, Q2, and Q3+Q4) with HR (95% CI) of 0.52 (0.11-2.40) for extreme categories.

**Abbreviations**: CVD, cardiovascular disease; OBS, oxidative balance score; Q, quartile; ref., reference.

**Multivariable model:** adjusted for age (underlying variable), family history of cardiovascular diseases (dichotomous), following special diet at baseline (dichotomous), marital status (married, single and others). Mediterranean adherence (continuous), prevalent cancer (dichotomous), prevalent depression (dichotomous), prevalent cardiovascular disease* (dichotomous), prevalent diabetes (dichotomous), prevalent dyslipidemia (dichotomous), prevalent hypertension (dichotomous), sex (dichotomous), total energy intake (continuous), use of aspirin (dichotomous), years of higher education (continuous), corresponding OBS components (dietary or lifestyle components), and stratified by deciles of age and recruitment period (6 categories).
*Prevalent cardiovascular disease was considered as having at least one of the following events before entering the cohort: aneurysm, angina pectoris, atrial fibrillation, cardiac insufficiency, coronary bypass, deep vein thrombosis, intermittent claudication, myocardial infarction, pulmonary embolism, stroke, or tachycardia

| **Table s5**. Associations of the items included in oxidative balance score (OBS) with all-cause, cancer, cardiovascular, and other-cause mortality in the SUN cohort (n = 18,561). Hazard ratios (HR) and 95% confidence intervals (CI). | | | | | | | |
| --- | --- | --- | --- | --- | --- | --- | --- |
|  | **Points of the Oxidative Balance Score (OBS)** | | | | | | |
|  | 0 points | 1 point | | 2 points | 3 points | | 4 points |
| **All-cause mortality** |  |  | |  |  | |  |
| ^1^Total Vitamin C (mg/d) | 1 (Ref.) | 0.83 (0.59-1.16) | | 0.85 (0.59-1.22) | 0.84 (0.57-1.26) | | 0.66 (0.41-1.06) |
| ^1^Total Vitamin E (mg/d) | 1 (Ref.) | 1.20 (0.88-1.64) | | 1.11 (0.80-1.53) | 1.20 (0.85-1.71) | | 1.03 (0.71-1.48) |
| Dietary Beta-carotenes (mcg/d) | 1 (Ref.) | 0.94 (0.69-1.28) | | 0.95 (0.69-1.31) | 0.85 (0.61-1.20) | | 0.89 (0.61-1.31) |
| Dietary Selenium (mcg/d) | 1 (Ref.) | 1.08 (0.78-1.48) | | 0.86 (0.60-1.25) | 0.89 (0.61-1.30) | | 0.99 (0.66-1.49) |
| ^1^Total Zinc (mg/d) | 1 (Ref.) | 0.89 (0.64-1.23) | | 0.84 (0.58-1.21) | 0.90 (0.62-1.30) | | 0.87 (0.61-1.25) |
| Dietary Heme Iron (mg/d) | 1 (Ref.) | 1.24 (0.87-1.77) | | 0.97 (0.67-1.41) | 0.99 (0.67-1.46) | | 1.25 (0.83-1.89) |
| ^2^Dietary PAC score | 1 (Ref.) | 1.34 (0.93-1.94) | | 1.35 (0.88-2.08) | 1.61 (1.02-2.55) | | 1.62 (0.94-2.79) |
| Dietary TAC (mmol Fe^+2^/100g) | 1 (Ref.) | 1.12 (0.76-1.64) | | 1.24 (0.82-1.87) | 1.11 (0.71-1.74) | | 0.82 (0.49-1.36) |
| BMI (kg/m^2^) | 1 (Ref.) | 0.65 (0.29-1.43) | | 0.59 (0.28-1.27) | 0.44 (0.20-0.94) | | 0.59 (0.28-1.26) |
| Alcohol Intake (g/d) | 1 (Ref.) | 0.45 (0.20-1.01) | | 0.25 (0.12-0.52) | 0.20 (0.09-0.42) | | 0.17 (0.08-0.35) |
|  | 0 points | | 2 points | | | 4points | |
| Smoking status | 1 (Ref.) | | 0.83 (0.64-1.08) | | | 0.67 (0.50-0.90) | |
| ^3^PA score | 1 (Ref.) | | 0.68 (0.54-0.84) | | | 0.65 (0.45-0.92) | |
|  |  |  | |  |  | |  |
| **CVD mortality** |  |  | |  |  | |  |
| ^1^Total Vitamin C (mg/d) | 1 (Ref.) | 0.63 (0.29-1.38) | | 0.65 (0.28-1.49) | 0.43 (0.16-1.15) | | 0.43 (0.14-1.35) |
| ^1^Total Vitamin E (mg/d) | 1 (Ref.) | 1.47 (0.68-3.18) | | 0.86 (0.36-2.03) | 1.69 (0.71-4.03) | | 1.62 (0.68-3.88) |
| Dietary Beta-carotenes (mcg/d) | 1 (Ref.) | 0.61 (0.29-1.29) | | 0.61 (0.28-1.32) | 0.48 (0.21-1.10) | | 0.61 (0.25-1.46) |
| Dietary Selenium (mcg/d) | 1 (Ref.) | 0.51 (0.24-1.08) | | 0.51 (0.22-1.16) | 0.28 (0.10-0.76) | | 0.43 (0.16-1.14) |
| ^1^Total Zinc (mg/d) | 1 (Ref.) | 1.14 (0.52-2.51) | | 1.01 (0.40-2.56) | 1.06 (0.44-2.55) | | 1.02 (0.44-2.34) |
| Dietary Heme Iron (mg/d) | 1 (Ref.) | 1.14 (0.46-2.79) | | 1.13 (0.46-2.75) | 0.48 (0.18-1.32) | | 0.75 (0.26-2.14) |
| ^2^Dietary PAC score | 1 (Ref.) | 2.26 (0.89-5.75) | | 3.16 (1.04-9.57) | 2.78 (0.87-8.89) | | 2.20 (0.51-9.40) |
| Dietary TAC (mmol Fe^+2^/100g) | 1 (Ref.) | 1.90 (0.72-5.01) | | 1.29 (0.45-3.70) | 1.48 (0.48-4.59) | | 1.27 (0.36-4.53) |
| BMI (kg/m^2^) | 1 (Ref.) | 1.15 (0.13-10.41) | | 1.02 (0.12-8.58) | 0.44 (0.05-3.79) | | 0.53 (0.06-4.45) |
| Alcohol Intake (g/d) | 1 (Ref.) | 0.35 (0.06-1.86) | | 0.11 (0.02-0.47) | 0.10 (0.02-0.50) | | 0.09 (0.02-0.40) |
|  | 0 points | | 2 points | | | 4points | |
| Smoking status | 1 (Ref.) | | 1.36 (0.65-2.84) | | | 0.68 (0.29-1.60) | |
| ^3^PA score | 1 (Ref.) | | 0.86 (0.51-1.44) | | | 0.17 (0.04-0.78) | |
|  |  | |  | | |  | |
| **Cancer mortality** |  |  | |  |  | |  |
| ^1^Total Vitamin C (mg/d) | 1 (Ref.) | 0.86 (0.54-1.39) | | 0.93 (0.56-1.54) | 0.95 (0.55-1.64) | | 0.61 (0.31-1.18) |
| ^1^Total Vitamin E (mg/d) | 1 (Ref.) | 1.37 (0.88-2.12) | | 1.49 (0.96-2.33) | 1.14 (0.69-1.88) | | 1.18 (0.70-1.97) |
| Dietary Beta-carotenes (mcg/d) | 1 (Ref.) | 0.88 (0.57-1.34) | | 0.84 (0.54-1.32) | 0.68 (0.41-1.11) | | 0.75 (0.44-1.28) |
| Dietary Selenium (mcg/d) | 1 (Ref.) | 1.44 (0.90-2.29) | | 1.20 (0.71-2.02) | 1.35 (0.79-2.30) | | 1.60 (0.90-2.87) |
| ^1^Total Zinc (mg/d) | 1 (Ref.) | 0.92 (0.59-1.42) | | 0.75 (0.45-1.24) | 0.81 (0.48-1.36) | | 0.79 (0.47-1.32) |
| Dietary Heme Iron (mg/d) | 1 (Ref.) | 1.26 (0.76-2.06) | | 1.11 (0.66-1.87) | 1.19 (0.70-2.04) | | 1.56 (0.88-2.77) |
| ^2^Dietary PAC score | 1 (Ref.) | 1.59 (0.92-2.74) | | 1.53 (0.83-2.83) | 2.11 (1.09-4.06) | | 2.48 (1.17-5.25) |
| Dietary TAC (mmol Fe^+2^/100g) | 1 (Ref.) | 1.18 (0.66-2.12) | | 1.66 (0.91-3.01) | 1.24 (0.65-2.36) | | 1.02 (0.50-2.11) |
| BMI (kg/m^2^) | 1 (Ref.) | 0.46 (0.15-1.44) | | 0.52 (0.18-1.51) | 0.46 (0.16-1.35) | | 0.55 (0.19-1.58) |
| Alcohol Intake (g/d) | 1 (Ref.) | 0.37 (0.11-1.19) | | 0.32 (0.11-0.88) | 0.22 (0.08-0.63) | | 0.18 (0.07-0.50) |
|  | 0 points | | 2 points | | | 4points | |
| Smoking status | 1 (Ref.) | | 0.65 (0.46-0.92) | | | 0.54 (0.36-0.80) | |
| ^3^PA score | 1 (Ref.) | | 0.74 (0.55-1.01) | | | 0.85 (0.53-1.34) | |
|  |  | |  | | |  | |
| **Other-cause mortality** |  |  | |  |  | |  |
| ^1^Total Vitamin C (mg/d) | 1 (Ref.) | 1.02 (0.54-1.91) | | 0.96 (0.47-1.98) | 1.28 (0.59-2.77) | | 1.30 (0.54-3.14) |
| ^1^Total Vitamin E (mg/d) | 1 (Ref.) | 0.82 (0.46-1.48) | | 0.69 (0.36-1.31) | 1.03 (0.54-1.96) | | 0.65 (0.32-1.33) |
| Dietary Beta-carotenes (mcg/d) | 1 (Ref.) | 1.46 (0.80-2.64) | | 1.44 (0.77-2.69) | 1.70 (0.91-3.20) | | 1.37 (0.65-2.87) |
| Dietary Selenium (mcg/d) | 1 (Ref.) | 1.04 (0.58-1.85) | | 0.55 (0.26-1.14) | 0.73 (0.35-1.50) | | 0.66 (0.30-1.47) |
| ^1^Total Zinc (mg/d) | 1 (Ref.) | 0.73 (0.38-1.40) | | 0.94 (0.48-1.83) | 0.97 (0.49-1.92) | | 0.86 (0.44-1.66) |
| Dietary Heme Iron (mg/d) | 1 (Ref.) | 1.29 (0.68-2.44) | | 0.64 (0.31-1.33) | 0.92 (0.45-1.87) | | 1.11 (0.53-2.35) |
| ^2^Dietary PAC score | 1 (Ref.) | 0.74 (0.39-1.40) | | 0.65 (0.29-1.45) | 0.66 (0.28-1.54) | | 0.63 (0.23-1.78) |
| Dietary TAC (mmol Fe^+2^/100g) | 1 (Ref.) | 0.84 (0.43-1.63) | | 0.79 (0.38-1.65) | 0.88 (0.40-1.96) | | 0.43 (0.16-1.15) |
| BMI (kg/m^2^) | 1 (Ref.) | 0.66 (0.16-2.66) | | 0.43 (0.11-1.69) | 0.31 (0.08-1.25) | | 0.53 (0.14-2.02) |
| Alcohol Intake (g/d) | 1 (Ref.) | 0.78 (0.14-4.38) | | 0.25 (0.05-1.35) | 0.23 (0.04-1.27) | | 0.23 (0.05-1.21) |
|  | 0 points | | 2 points | | | 4points | |
| Smoking status | 1 (Ref.) | | 0.99 (0.58-1.69) | | | 0.92 (0.52-1.62) | |
| ^3^PA score | 1 (Ref.) | | 0.48 (0.32-0.73) | | | 0.59 (0.31-1.11) | |

**Abbreviations:** CVD, cardiovascular disease; OBS, oxidative balance score; ref., reference.

^1^Total accounts for intakes from both dietary and supplemental sources.

^2^Score proposed by Pounis et al. (29)

^3^Score proposed by Alvarez-Alvarez et al. (38)
**Multivariable model:** adjusted for age (underlying variable), family history of cardiovascular diseases (dichotomous), following special diet at baseline (dichotomous), marital status (married, single and others). Mediterranean adherence (continuous), prevalent cancer (dichotomous), prevalent depression (dichotomous), prevalent cardiovascular disease* (dichotomous), prevalent diabetes (dichotomous), prevalent dyslipidaemia (dichotomous), prevalent hypertension (dichotomous), sex (dichotomous), total energy intake (continuous), use of aspirin (dichotomous), years of higher education (continuous), and stratified by deciles of age and recruitment period (6 categories). Additionally, the model was mutually adjusted for the other components of the OBS.
*Prevalent cardiovascular disease was considered as having at least one of the following events before entering the cohort: aneurysm, angina pectoris, atrial fibrillation, cardiac insufficiency, coronary bypass, deep vein thrombosis, intermittent claudication, myocardial infarction, pulmonary embolism, stroke, or tachycardia.

**FIGURES**

**A)**  **B)**


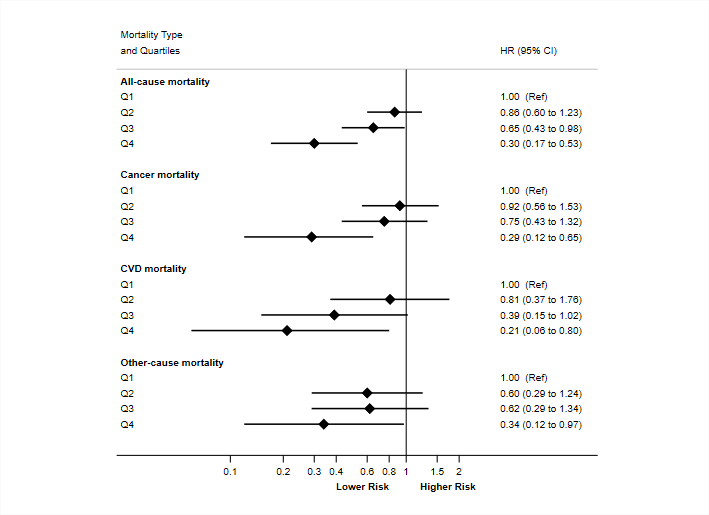
**
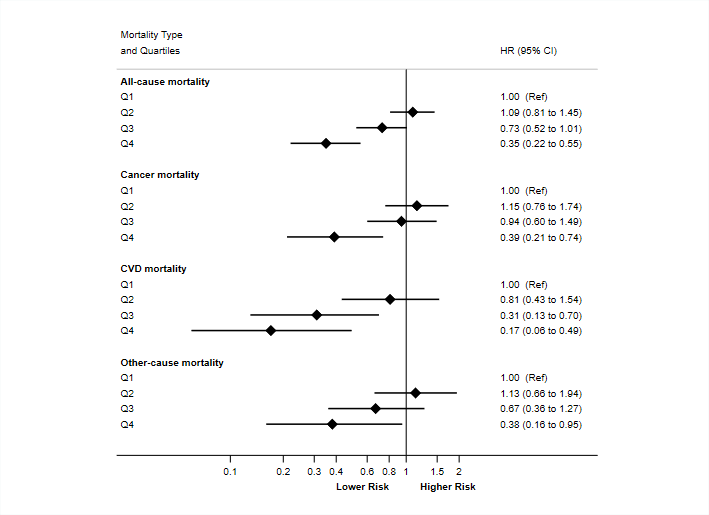
**


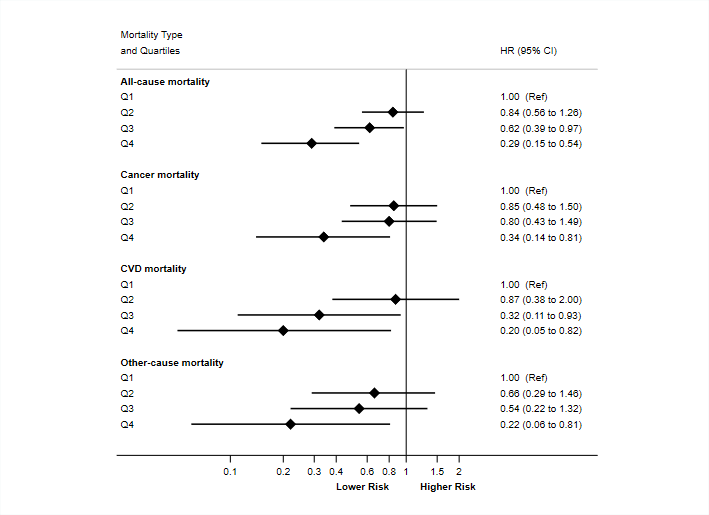
**C)**  **D)**


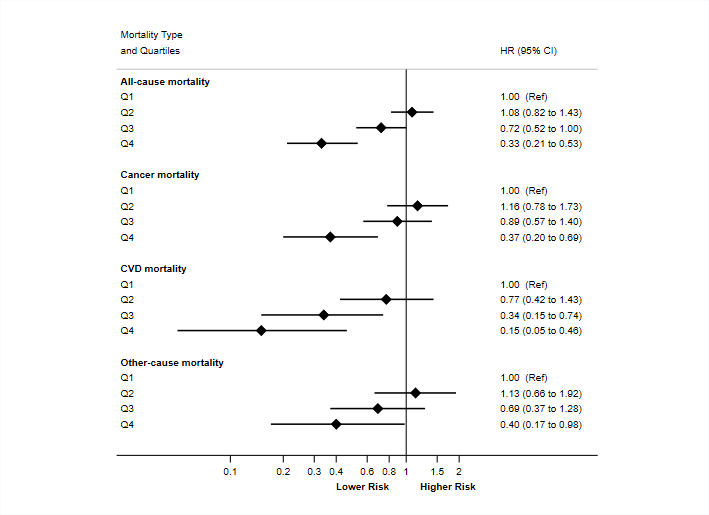


**E) F)**

**
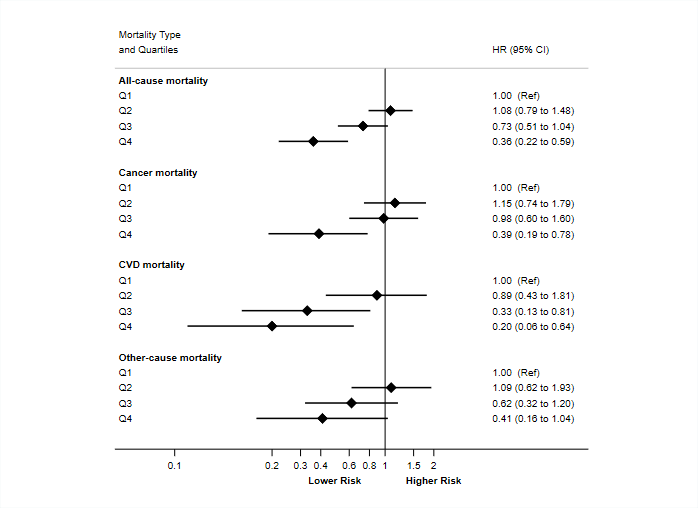
**

**
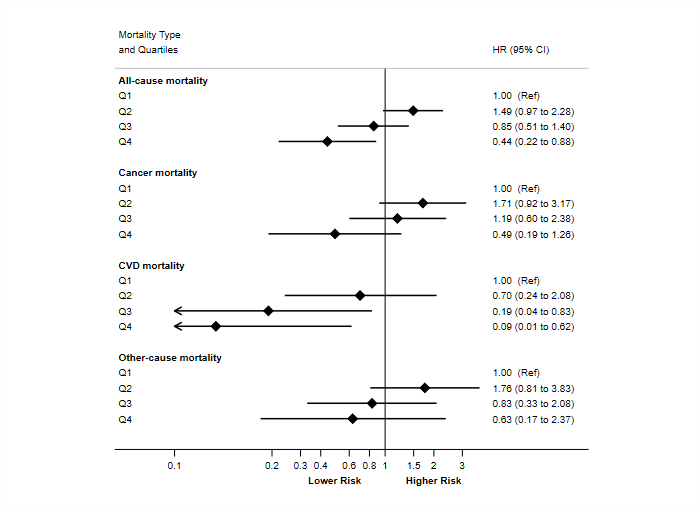
**

**
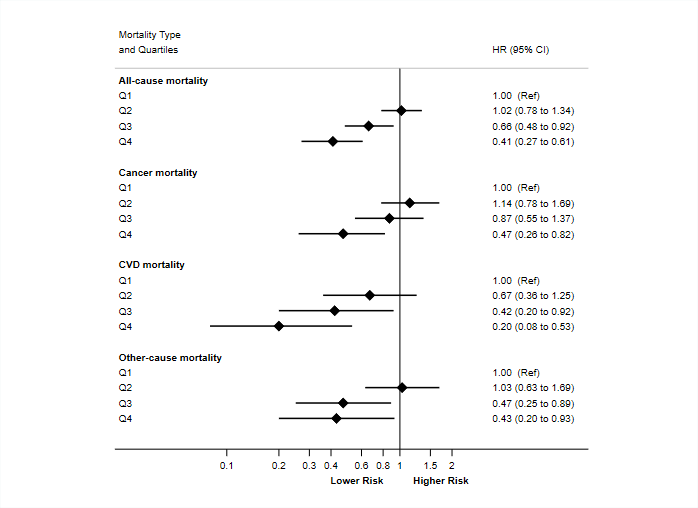
G)**

**Figure s1.** Sensitivity analyses. Hazard Ratio (HR) and 95% Confidence Interval (CI) for the associations of the oxidative balance score (OBS) with all-cause, cancer, cardiovascular, and other-cause mortality under different assumptions: **A**) excluding participants with < 2years of follow-up (n= 18,082); **B**) truncating the participants’ follow-up at 10 years (n= 18,561); **C**) excluding participants with < 2 years of follow-up and truncating the follow-up at 10 years (n=18,082); **D)** excluding participants with < 40 years at the end of the follow-up (n=13,881); **E)** excluding participants with < 4 years of follow-up (n= 17,105); **F)** excluding participants with < 10 years of follow-up (n= 12,500); **G)** re-calculating the OBS according to total vitamin C, total vitamin E, beta-carotenes, selenium, total zinc, and heme iron sex-specific quintile values (n=18,561), in the SUN cohort (1999–2018).

**Acknowledgments**

We thank other members of the SUN Group: Aguilera-Buenosvinos I, Alonso A, Álvarez-Álvarez I, Balaguer A, Barbería-Latasa M, Barrio-López MT, Basterra-Gortari FJ, Battezzati A, Bazal P, Benito S, Bertoli S, Bes-Rastrollo M, Beunza JJ, Buil-Cosiales P, Carlos S, de Irala J, de la Fuente-Arrillaga C, de la O V, de la Rosa PA, Delgado-Rodríguez M, Díaz-Gutiérrez J, Díez Espino J, Domínguez L, Donat-Vargas C, Donazar M, Eguaras S, Fernández-Lázaro CI, Fernández-Montero A, Fresán U, Galbete C, García-Arellano A, Gardeazábal I, Gea A, Gutiérrez-Bedmar M, Gomes-Domingos AL, Gómez-Donoso C, Gómez-Gracia E, Goñi E, Goñi L, Guillén F, Hernández-Hernández A, Hershey MS, Hidalgo-Santamaría M, Hu E, Lahortiga F, Leone A, Llavero M, Llorca J, López del Burgo C, Marí A, Martí A, Martín-Calvo N, Martín-Moreno JM, Martínez JA, Martínez-González MA, Mendonça R, Menéndez C, Molendijk M, Molero P, Muñoz M, Navarro AM, Pano O, Pérez de Ciriza P, Pérez-Cornago A, Pérez de Rojas J, Pimenta AM, Ramallal R, Razquin C, Rico-Campà A, Romanos-Nanclares A, Ruiz L, Ruiz-Canela M, San Julián B, Sánchez D, Sánchez-Bayona R, Sánchez-Tainta A, Sánchez-Villegas A, Santiago S, Sayón-Orea C, Toledo E, Vázquez Z, Zazpe I.
